# Comparison of the Differential Diagnostic Performance of Intravoxel Incoherent Motion Imaging and Diffusion Kurtosis Imaging in Malignant and Benign Thyroid Nodules

**DOI:** 10.3389/fonc.2022.895972

**Published:** 2022-07-22

**Authors:** Liling Jiang, Jiao Chen, Haiping Huang, Jian Wu, Junbin Zhang, Xiaosong Lan, Daihong Liu, Jiuquan Zhang

**Affiliations:** ^1^ Department of Radiology, Cancer Hospital, Chongqing University, Chongqing, China; ^2^ Department of Pathology, Cancer Hospital, Chongqing University, Chongqing, China; ^3^ Head and Neck Cancer Center, Cancer Hospital, Chongqing University, Chongqing, China

**Keywords:** magnetic resonance imaging, intravoxel incoherent motion, diffusion kurtosis imaging, thyroid nodules, Ki-67

## Abstract

**Objective:**

This study aimed to compare the diagnostic capacity between IVIM and DKI in differentiating malignant from benign thyroid nodules.

**Material and Methods:**

This study is based on magnetic resonance imaging data of the thyroid with histopathology as the reference standard. Spearman analysis was used to assess the relationship of IVIM-derived parameters D, f, D* and the DKI-derived parameters D_app_ and K_app_. The parameters of IVIM and DKI were compared between the malignant and benign groups. Binary logistic regression analysis was performed to establish the diagnostic model, and receiver operating characteristic (ROC) curve analysis was subsequently performed. The DeLong test was used to compare the diagnostic effectiveness of different prediction models. Spearman analysis was used to assess the relationship of Ki-67 expression and parameters of IVIM and DKI.

**Results:**

Among the 93 nodules, 46 nodules were malignant, and 47 nodules were benign. The D_app_ of DKI-derived parameter was related to the D (P < 0.001, r = 0.863) of IVIM-derived parameter. The K_app_ of DKI-derived parameter was related to the D (P < 0.001, r = -0.831) of IVIM-derived parameters. The malignant group had a significantly lower D value (P < 0.001) and f value (P = 0.013) than the benign group. The malignant group had significantly higher K_app_ and lower D_app_ values (all P < 0.001). The D+f had an area under the curve (AUC) of 0.951. The D_app_+K_app_ had an AUC of 0.943. The D+f+D_app_+K_app_ had an AUC of 0.954. The DeLong test showed no statistical significance among there prediction models. The D (P = 0.007) of IVIM-derived parameters and D_app_ (P = 0.045) of DKI-derived parameter were correlated to the Ki-67 expression.

**Conclusions:**

IVIM and DKI were alternative for each other in in differentiating malignant from benign thyroid nodules.

## Introduction

Thyroid nodules are common in adults. It was reported that incidental thyroid nodules are found on 20% to 67% of ultrasound examinations, up to 25% of contrast-enhanced thoracic computed tomography scans, 16% to 18% of magnetic resonance imaging (MRI) scans, and 1% to 2.3% of positron emission tomography scans (1). Due to avoiding unnecessary surgery, effective diagnostic methods that can provide reliable differentiation between malignant and benign thyroid nodules are urgently needed.

Ultrasound is widely used to detect thyroid nodules and in guidelines for biopsy and clinical therapy, and ultrasound-guided fine needle aspiration biopsy is valuable for the diagnosis of thyroid nodules. Nevertheless, up to 30% of fine needle aspiration biopsies show indeterminate cytology because of the finiteness of the puncture tissue (2). Computed tomography has been used to depict the relationship of the nodule with surrounding structure and lymph node metastasis. However, it is difficult to distinguish between benign and malignant thyroid nodules and poses radiation hazards (3). With the development of MRI techniques, diffusion weighted imaging (DWI) has become a promising modality for thyroid examination in recent years.

Intravoxel incoherent motion (IVIM) and diffusion kurtosis imaging (DKI), as diffusion derivative technology, have shown tremendous clinical potential in thyroid nodules (4–7). IVIM can separate the incoherent motion of water molecules within capillaries from extravascular molecular diffusion (8). IVIM theory could resolve pure diffusion coefficient (D), and perfusion related incoherent microcirculation (D*), separately, while also identifying the microvascular volume fraction (f). DKI can provide a more accurate model of diffusion and capture the non-Gaussian diffusion parameters for tissue heterogeneity (9). DKI theory could resolve the non-Gaussian diffusion coefficient (D_app_), and the apparent kurtosis coefficient (K_app_). Past study showed IVIM and DKI provide a more comprehensive description of tissue properties compared to DWI in thyroid (4, 5). However, the differential diagnostic capacity of IVIM and DKI remains to be revealed in patients with thyroid nodules.

Diffusion parameters were considered to reflect the cell size and density, extracellular space and intracellular architecture, which limits the cellular movement of water. Ki-67 is a cell proliferation protein that was related to cell density and extracellular space (10). It is unknown about the relationship between Ki-67 expression and the parameters of IVIM and DKI in thyroid papillary carcinoma.

In this study, we compared the diagnostic performance of IVIM and DKI to differentiate malignant nodules from benign thyroid nodules. In addition, we explored the relationship of Ki-67 expression and parameters of IVIM and DKI in thyroid papillary carcinoma.

## Materials and Methods

### Patient Collection and Thyroid Nodule Selection

This prospective study was approved by the local institutional review board (IRB No. CZLS2021207-A), and written informed consent was obtained from each patient. The study was conducted in accordance with the Declaration of Helsinki (as revised in 2013). From July 2020 and February 2021, 86 consecutive patients were recruited and underwent thyroid MRI examinations in this study.

The inclusion criterion was as follows: a) no contraindications for MRI examination; b) thyroid nodule excision was planned; c) patients had not needle biopsy or therapy before MRI scan; d) only the biggest nodule was selected when there were more than two nodules in one lobule. The exclusion criterion was as follows: a) IVIM or DKI had worse image quality; b) the nodules were cystic.

### Sample Size

There are no generally accepted approaches to estimate the sample size requirements for derivation and validation studies of prediction models, however, we ensured that the study met suggested requirements of having at least 10 events per candidate variable for the derivation of a model and at least 100 events for validation studies.

### Examination Method

MRI was performed on a 3.0 T whole body MRI system (SOMATOM Prisma, Siemens Healthineers, Forchheim, Germany) using a third-party 16 channel surface coil (Zhongzhi Medical, Jiangsu, China). The MRI protocol mainly included coronal fast dixion, axial T1 weighted imaging with fat suppression, axial T2 weighted imaging with fat suppression, axial IVIM and DKI. IVIM and DKI were performed using the ZOOMit technique with selective excitation based on parallel radio frequency pulses. The detailed protocol parameters are shown in **Table 1**.

**Table 1:** Imaging parameters of IVIM and DKI.

	IVIM	DKI
TR	4200 ms	4600 ms
TE	72 ms	72 ms
FOV	160 × 58 mm^2^	160 × 58 mm^2^
Average	2, 2, 2, 2, 4, 6, 9	1, 4, 6, 9
b-value	0, 50, 100, 200, 400, 600, 800 (×10^-3^ s/mm^2^)	0, 500, 1000, 1500 (×10^-3^ s/mm^2^)
Matrix size	36 × 110	36 × 110
Thickness	3 mm	3 mm
Intersection gap	0.3 mm	0.3 mm
Examination time	329 s	273 s

IVIM, intravoxel incoherent motion imaging; DKI, diffusion kurtosis imaging; TR, time repetition; TE, time echo; FOV, field of view.

### Image Analysis

All IVIM and DKI images were transferred to a workstation (syngo. via Frontier, Siemens Healthineers, Germany) for analysis. IVIM and DKI parameters were measured by observer 1 (LJ, with six years of experience in thyroid imaging). The IVIM and DKI parameters of the first 75 nodules were assigned by Observer 2 (JC, with two years of experience in thyroid imaging). The observers were blind to the surgical pathological results. The measurement was repeated one month later by observer 2. Regions of interest (ROI) was freehand drawn along the nodule margin in the largest slice to obtain relevant parameters. ROIs of IVIM and DKI were in the same slice, avoiding cystic, vessel and hemorrhage. The parameters derived from the IVIM were obtained through the previously reported fitting equation: S_b_/S_0_ = (1 - f) exp (-b D) + f exp (-b D*) (11). The following parameters were calculated: D, D* and f. The parameters derived from the DKI were obtained through the previously reported fitting equation: ${\text{S}}_{\text{b}}={\text{S}}_{0}\text{exp}\left(-b{\text{ D}}_{\text{app}}+\;b^{2}D_{\text{app}}^{2}{\text{K}}_{\text{app}}/6\right)$ (12). The following parameters were calculated: K_app_ and D_app_. The mean value was recorded for each ROI.

### Histopathologic Examination

According to the established convention, histopathologic examination was used as reference standards. Tissue samples of each nodule were obtained from operation. Surgically resected nodules were subjected to intra-operative frozen section analysis for preliminary risk assessment. The final diagnosis was based on postoperative paraffin section pathological examinations. In the event of suspicious malignant samples or atypical samples, immunohistochemical staining was applied to differentiate benign and malignant nodules. All diagnoses were determined by experienced pathologist. According to the pathological results, the nodules were assigned to either the benign or malignant groups.

### Statistical Analysis

Statistical analyses were performed using SPSS version 25.0, GraphPad Prism 7.0, and R version 4.0.1. The results were considered to be statistically significant at P < 0.05. The interclass correlation coefficient (ICC) was used to test the interobserver reliability and intraobserver reliability. The ICC was interpreted as follows: 0.00–0.20, poor correlation; 0.21–0.40, fair correlation; 0.41–0.60, moderate correlation; 0.61–0.80, good correlation; and 0.81–1.00, excellent correlation. The Kolmogorov–Smirnov test was performed to analyze normality. According to the results of the Kolmogorov–Smirnov test, Pearson analysis or Spearman analysis was used to assess the relationship of parameters of IVIM and DKI. The independent Student’s t test or Mann–Whitney U test were applied to check whether there were significant differences between the malignant and benign groups. When P < 0.05, variables were entered into binary logistic regression analysis. Single parameters and the combination of parameters used to establish the diagnostic model. Receiver operating characteristic (ROC) curve was applied to predict malignant nodules. The DeLong test was used to compare the diagnostic effectiveness of the prediction models. Pearson analysis or spearman analysis was used to assess the relationship of Ki-67 expression and parameters of IVIM and DKI in thyroid papillary carcinoma.

## Results

### Clinical Data

Of the 86 patients, 7 patients were excluded (2 patients with worse image quality and 5 patients with cystic nodules). Ultimately, among 79 patients with 93 nodules, 59 were female and 20 was males, with ages ranging from 21 to 77 years old. In the malignant group, 26 were female and 14 was males, with ages ranging from 21 to 67 years old. In the benign group, 33 were female and 6 were male, with ages ranging from 21 to 77 years old. There were 46 malignant nodules (44 papillary carcinoma, 1 follicular carcinoma, 1 medullary carcinoma), and 47 benign nodules (24 adenomas, 19 nodular goiters, 2 goiters with adenomatous hyperplasia, 1 subacute thyroiditis, 1 granulomatous inflammation). In the sample, 31patients with papillary carcinoma had the results of Ki-67.

### Relationship of Parameters of IVIM and DKI

**Figure 1:**
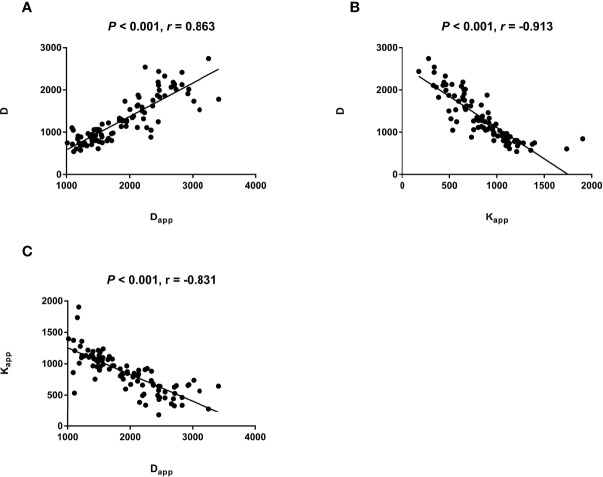
The scatter plot reflecting correlation of quantification parameters between IVIM and DKI.

The reproducibility of the two ROIs for measuring thyroid nodules with IVIM and DKI parameters is summarized in **Table 2**, with good ICC values. The D_app_ of DKI-derived parameter was correlated to the D (P < 0.001, r = 863) of IVIM-derived parameter (**Table 3**; **Figure 1**). The K_app_ of DKI-derived parameter was correlated to the D (P < 0.001, r = -0.831) of IVIM-derived parameters (**Table 3**, **Figure 1**).

**Table 2:** The interobserver and intraobserver reproducibility of measurements of thyroid nodules with IVIM and DKI.

	Interobserver (95% CI)	Intraobserver (95% CI)
D	0.865 (0.794-0.912)	0.928 (0.88-0.954)
D*	0.795 (0.694-0.865)	0.963 (0.943-0.977)
f	0.799 (0.700-0.868)	0.856 (0.781-0.907)
D_app_	0.872 (0.805-0.917)	0.957 (0.955-0.973)
K_app_	0.891 (0.833-0.930)	0.954 (0.963-0.985)

IVIM, intravoxel incoherent motion imaging; DKI, diffusion kurtosis imaging; CI, confidence interval; D, true diffusion coefficient; D*, pseudodiffusion coefficient; f, perfusion fraction; D_app_, apparent diffusion coefficient derived from DKI; K_app_, apparent diffusion kurtosis coefficient.

**Table 3:** The correlation analysis of quantification parameters of IVIM and DKI.

	*r*	*P*
D_app_ VS D	0.863	< 0.001
D_app_ VS D*	0.079	0.450
K_app_ VS D	-0.913	< 0.001
K_app_ VS D*	-0.049	0.643
K_app_ VS f	-0.079	0.447
D_app_ VS K_app_	-0.831	< 0.001
D VS D*	0.126	0.230
D VS f	0.060	0.569
D* VS f	0.137	0.190

IVIM, intravoxel incoherent motion imaging; DKI, diffusion kurtosis imaging; D, true diffusion coefficient; D*, pseudodiffusion coefficient; f, perfusion fraction; D_app_, apparent diffusion coefficient derived from DKI; K_app_, apparent diffusion kurtosis coefficient.

### Comparison of IVIM and DKI Parameters Between the Malignant and Benign Groups

**Figure 2:**
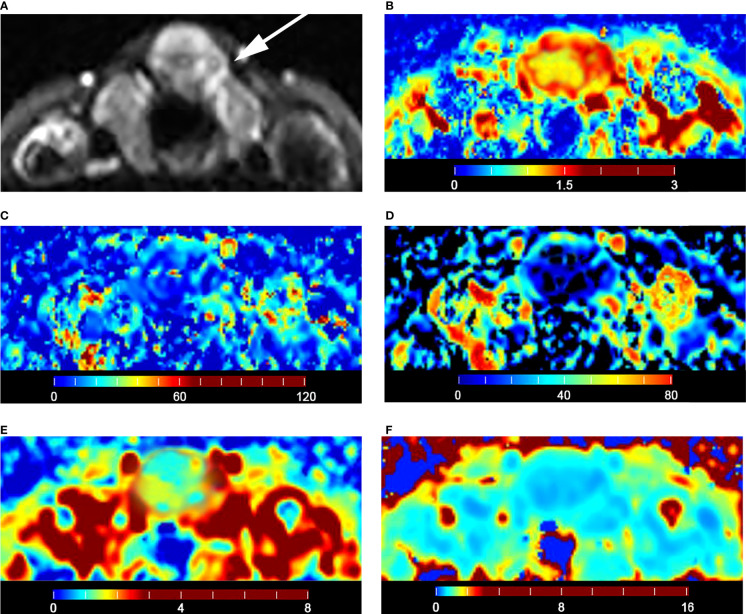
Images of a 36-year-old female with isthmus papillary carcinoma: **(A)** axial diffusion image with b = 0; **(B)** color map of D, D value of lesion is 0.93 × 10^-3^ mm^2^/s; **(C)** color map of D*, D* value of lesion is 16.04 × 10^-3^ mm^2^/s; **(D)** color map of f, f value of lesion is 14.21%; **(E)** color map of D_app_, D_app_ value of lesion is 1.53 × 10^-3^ mm^2^/s; **(F)** color map of K_app_, K_app_ value of lesion is 1.05.

**Figure 3:**
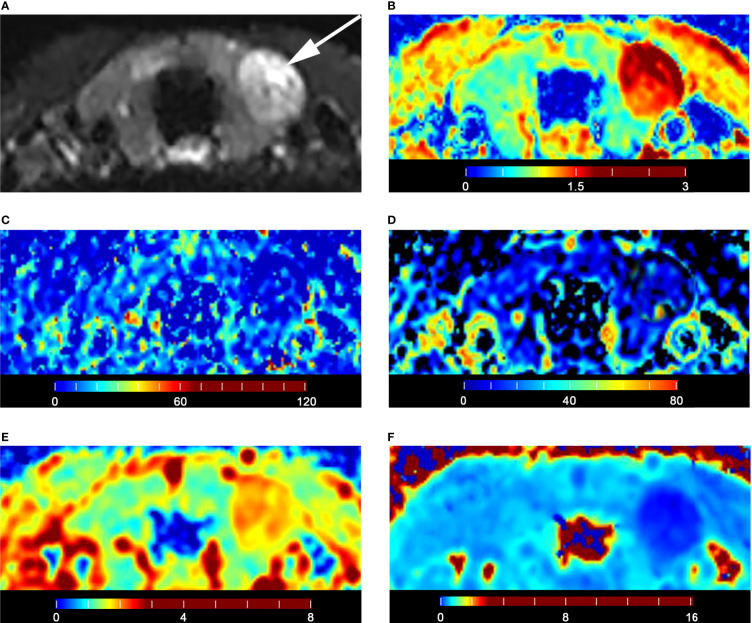
Images of a 28-year-old male with left lobe follicular adenoma: **(A)** axial diffusion image with b = 0; **(B)** color map of D, D value of lesion is 1.79 × 10^-3^ mm^2^/s; **(C)** color map of D*, D* value of lesion is 15.74 × 10^-3^ mm^2^/s; **(D)** color map of f, f value of lesion is 18.35%; **(E)** color map of D_app_, D_app_ value of lesion is 2.06 × 10^-3^ mm^2^/s; **(F)** color map of K_app_, K_app_ value of lesion is 0.50.

The malignant group had a significantly lower D value (P < 0.001) and f value (P = 0.013) than the benign group (**Table 4**). The D* value was not significantly different between malignant and benign nodules (P = 0.666) (**Table 4**). The malignant group had a significantly higher K_app_ (P < 0.001) and lower D_app_ (P < 0.001) (**Table 4**). The color maps of IVIM- and DKI-derived parameters were shown in **Figures 2**, **3**.

**Table 4:** Quantification parameters of IVIM and DKI for the benign and malignant groups.

	Benign group (n = 47)	Malignant group (n = 46)	*P*
D (×10^-3^ mm^2^/s)	1.71 ± 0.48	0.92 ± 0.22	< 0.001
D* (×10^-3^ mm^2^/s)	16.56 ± 4.76	16.08 ± 5.92	0.666
f (%)	18.37 ± 0.83	14.54 ± 0.60	0.013
D_app_ (×10^-3^ mm^2^/s)	2.37 ± 0.46	1.48 ± 0.27	< 0.001
K_app_	0.65 ± 0.26	1.07 ± 0.22	< 0.001

IVIM, intravoxel incoherent motion imaging; DKI, diffusion kurtosis imaging; D, true diffusion coefficient; D*, pseudodiffusion coefficient; f, perfusion fraction; D_app_, apparent diffusion coefficient derived from DKI; K_app_, apparent diffusion kurtosis coefficient.

### Diagnostic Performance Evaluation

**Figure 4:**
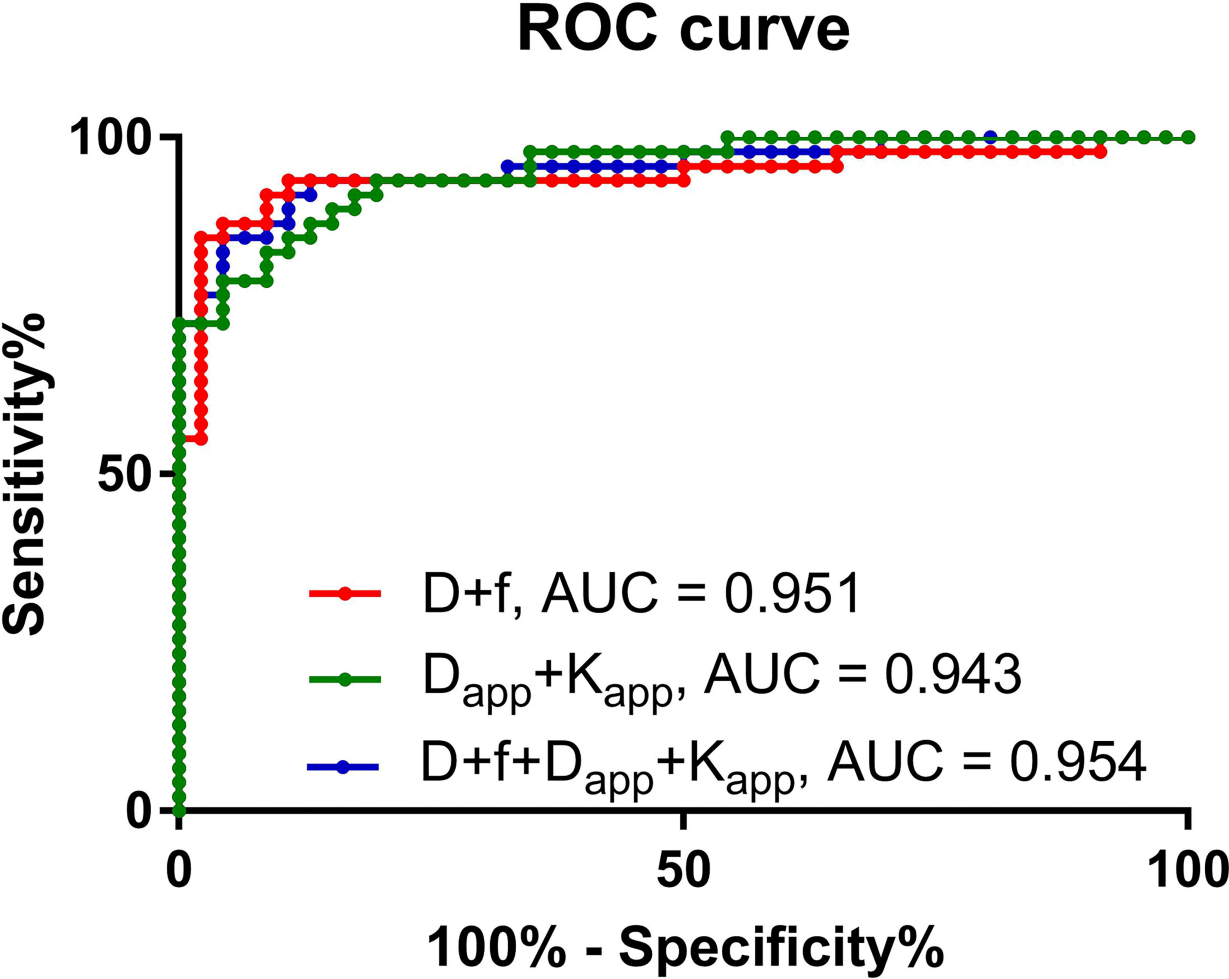
Receiver operating characteristic curve of the predictive performance of different prediction models.

Among the quantification parameters of IVIM, the D value had an area under the curve (AUC) of 0.928, the f value had an AUC of 0.637, and D+f had an AUC of 0.951 in predicting thyroid malignant nodules (**Table 5**). In the quantification parameters of DKI, D_app_ had an AUC of 0.943, K_app_ had an AUC of 0.921, and D_app_+K_app_ had an AUC of 0.943 in predicting malignant thyroid nodules (**Table 5**). D+f+D_app_+K_app_ had the highest AUC of 0.954 (**Table 5**). However, the DeLong test shows that there was no statistically significant difference among D+f, D_app_+K_app_ and D+f+D_app_+K_app_ (**Table 6**) (**Figure 4**).

**Table 5:** The diagnostic performance of the parameters of IVIM and DKI.

	True positive	False positive	Sensitivity	Specificity	AUC
D	91.30%	8.70%	87.23%	93.48%	0.928
f	63.04%	36.96%	73.91%	53.57%	0.637
D+f	89.13%	10.87%	82.98%	91.30%	0.951
D_app_	91.30%	8.70%	91.30%	91.49%	0.943
K_app_	86.96%	13.04%	93.48%	82.98%	0.921
D_app_+K_app_	89.13%	10.87%	87.23%	95.65%	0.943
D+f+D_app_+K_app_	89.13%	10.87%	93.62%	86.96%	0.954

IVIM, intravoxel incoherent motion imaging; DKI, diffusion kurtosis imaging; AUC, area under the curve; D, true diffusion coefficient; f, perfusion fraction; D_app_, apparent diffusion coefficient derived from DKI; K_app_, apparent diffusion kurtosis coefficient.

**Table 6:** Comparison of the diagnostic performance between IVIM and DKI for differential diagnosis by DeLong test.

	*P*
D+f VS D_app_+K_app_	0.623
D_app_+K_app_+D+f VS D+f	0.736
D_app_+K_app_+D+f VS D_app_+K_app_	0.325

IVIM, intravoxel incoherent motion imaging; DKI, diffusion kurtosis imaging; D, true diffusion coefficient; f, perfusion fraction; D_app_, apparent diffusion coefficient derived from DKI; K_app_, apparent diffusion kurtosis coefficient.

### Relationship of Ki-67 Expression and Quantification Parameters of IVIM and DKI in Thyroid Papillary Carcinoma

**Figure 5:**
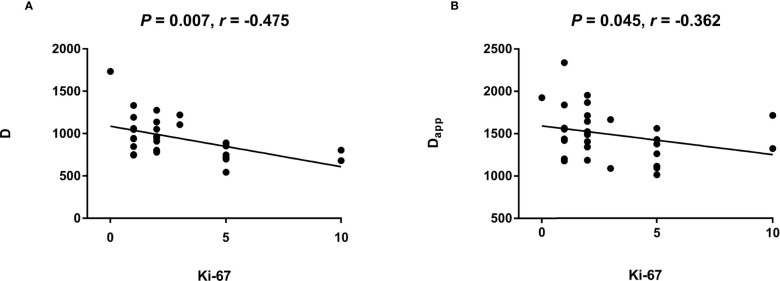
The scatter plot reflecting correlation between Ki-67 and quantification parameters of IVIM and DKI.

**Figure 6:**
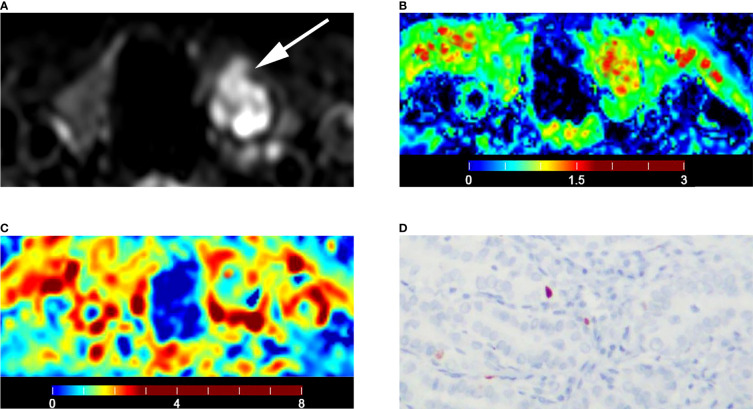
Images of a 55-year-old male with left lobe papillary carcinoma (A-D): **(A)** axial diffusion image with b = 0; **(B)** color map of D, D value of lesion is 1.06 × 10^-3^ mm^2^/s; **(C)** color map of D_app_, D_app_ value of lesion is 1.43 × 10^-3^ mm^2^/s; **(D)** Ki-67 expression is 1% in tumor cells.

**Figure 7:**
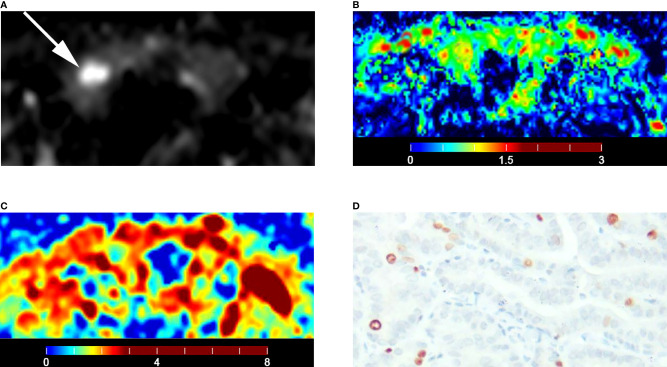
Images of a 51-year-old female with right lobe papillary carcinoma (A-D): **(A)** axial diffusion image with b = 0; **(B)** color map of D, D value of lesion is 0.70 × 10^-3^ mm^2^/s; **(C)** color map of D_app_, D_app_ value of lesion is 1.26 × 10^-3^ mm^2^/s; **(D)** Ki-67 expression is 5% in tumor cells.

The D (P = 0.007) of IVIM-derived parameters was related to the Ki-67 expression (**Table 7**) (**Figure 5**). The D_app_ (P = 0.045) of DKI-derived parameter was related to the Ki-67 expression (**Table 7**) (**Figure 5**). Representative D maps, D_app_ maps and immunohistochemical staining pictures (**Figures 6**, **7**) were illustrated.

**Table 7:** The correlation analysis between Ki-67 and quantification parameters of IVIM and DKI in thyroid papillary carcinoma.

	*r*	*P*
D	-0.475	0.007
D*	0.042	0.824
f	-0.079	0.672
D_app_	-0.362	0.045
K_app_	0.284	0.122

IVIM, intravoxel incoherent motion imaging; DKI, diffusion kurtosis imaging; D, true diffusion coefficient; D*, pseudodiffusion coefficient; f, perfusion fraction; D_app_, apparent diffusion coefficient derived from DKI; K_app_, apparent diffusion kurtosis coefficient.

## Discussion

It is necessary to differentiate malignant from benign lesions when detecting thyroid nodules using imaging methods. In this study, the DKI-derived parameters were related to the IVIM-derived parameters. The malignant and benign nodules exhibited significantly different D, f, D_app_ and K_app_ values. Moreover, these parameters of IVIM and DKI had comparable differential diagnostic ability for thyroid nodules. Combination IVIM with DKI cannot improve diagnostic performance. In addition, both DKI-derived parameters and IVIM-derived parameters were related with Ki-67 expression.

IVIM is a noninvasive technique that shows superiority in reflecting tumor cellularity and perfusion without the need for contrast agents. It has already been applied in the differentiation of lung nodules (13), thyroid nodules (14), prostate (15) and brain tumors (16) with good diagnostic performance. In our study, malignant nodules demonstrated lower D values than did benign nodules, which is consistent with previous studies (14, 17). The D value can precisely reflect the true diffusion without the influence of perfusion-related diffusion, which can calculated with the data from b-values higher than 200 mm^2^/s using a monoexponential model (18). In addition, malignant nodules demonstrated a significantly lower f value than did benign nodules, but the f value had undesirable differential diagnostic capacity. The f value mainly reflects pseudodiffusion. When the b-value is lower than 200 mm^2^/s, the data are fitted to a biexponential model to acquire the f value (19). The ZOOMit technique restricts the b-value to multiples of 50. Therefore, the b-value cannot be set to 10 and 90 mm^2^/s, resulting in the worse differentiation performance. In this study, the D* value was not significantly different between benign and malignant nodules. D* reflects the contribution of perfusion to signal attenuation of the diffusion image (20). It has been reported that the D* value may be unreliable in the IVIM model for differential diagnosis and has poor measurement reproducibility (21).

DKI is a non-Gaussian diffusion weighted analysis method. The diffusion of water molecules in the microenvironment deviates from the mono-exponential Gaussian model at high b-values, leading to inaccurate fitting and poor calculation of the diffusion coefficient. Considering the above factors, a DKI model was developed and showed good diagnostic performance for prostate cancer (22), hepatocellular carcinoma (23), and glioma (24). It has been reported that DKI-derived parameters demonstrated an advantage compared to conventional DWI for thyroid lesion diagnosis (4). Certainly, in this study, there were significant differences in D_app_ and K_app_ values between the benign and malignant nodules, consistent with a previous study (4). In the DKI model, restricted diffusion is due to cell proliferation and increases in cellularity, consequently, a reduction in extracellular and intercellular spaces. D_app_ was correlated with extracellular changes, and K_app_ was more sensitive to the intracellular architecture (25). This technique allows for the measurement of the excess diffusion kurtosis of the tissue while quantifying the deviation of tissue diffusion from the standard Gaussian pattern (26). In this respect, this technique is expected to more accurately reflect the microstructural complexity of human tissue (27). Malignant thyroid nodules have lower D_app_ and higher K_app_ values than benign nodules, which means that malignant nodules may have less extracellular space and a tighter intracellular architecture.

Both DKI-derived parameters and IVIM-derived parameters were related with Ki-67 expression. In pathological studies, proliferative activity of cells is often evaluated by the expression of Ki-67, one of the most common proliferation markers that is expressed in all active phases of the cell cycle (28). The D value can precisely reflect the true diffusion without the influence of perfusion-related diffusion. The expression of Ki-67 lead to restricted diffusion, which could result in low D value. In addition, rapid cell proliferation could lead to less extracellular space, which could result in low D_app_. The strong linear relationship between IVIM-derived parameters and DKI-derived parameters led to the alternative for each other in in differentiating malignant from benign thyroid nodules.

This study does have some limitations. First, the ZOOMit technique restricts the b-value to multiples of 50; otherwise, the f value may have better diagnostic performance. Second, only binary logistic regression was used to build the prediction model. Other statistical models should be introduced in subsequent work. Third, the number of Ki-67 cases is relatively small. A larger patient cohort is needed in future studies to confirm the present results.

## Conclusions

IVIM and DKI showed comparable differential diagnostic capacity in differentiating malignant from benign nodules. Combination IVIM with DKI cannot improve diagnostic performance. The D of IVIM-derived parameters and D_app_ DKI-derived parameter was related to the Ki-67 expression in thyroid papillary carcinoma. In conclusion, IVIM and DKI were alternative for each other in in differentiating malignant from benign thyroid nodules.

## Data Availability Statement

The raw data supporting the conclusions of this article will be made available by the authors, without undue reservation.

## Ethics Statement

The studies involving human participants were reviewed and approved by Chongqing University Cancer Hospital institutional review board. The patients/participants provided their written informed consent to participate in this study.

## Author’s Contributions

JW contributed to the conception and design of the study, data analysis and writing of the manuscript. LJ and DL contributed to performing the experiments and writing and revising the manuscript. JC, JZ and ML contributed to the data collection. DL, XL and HH contributed to the data analysis and interpretation of the data. JZ is the guarantor of this study and approved the version to be submitted. All authors accept responsibility for the integrity of the data and the accuracy of the data analysis. All authors read and approved the final manuscript.

## Funding

This study has received funding by the National Natural Science Foundation of China (Grant No. 82071883), combination projects of medicine and engineering of the Fundamental Research Funds for the Central Universities in 2019 (project No. 2019CDYGYB008), 2020 SKY Imaging Research Fund of the Chinese International Medical Foundation (project No. Z-2014-07-2003-24). The authors declare that this study received the support of Zhitao Zhang from Siemens Healthcare, Ltd., Chengdu Branch. The funder was not involved in the study design, collection, analysis, interpretation of data, the writing of this article or the decision to submit it for publication.

## Conflict of Interest

The authors declare that the research was conducted in the absence of any commercial or financial relationships that could be construed as a potential conflict of interest.

## Publisher’s Note

All claims expressed in this article are solely those of the authors and do not necessarily represent those of their affiliated organizations, or those of the publisher, the editors and the reviewers. Any product that may be evaluated in this article, or claim that may be made by its manufacturer, is not guaranteed or endorsed by the publisher.

## Abbreviations

AUC, Area under the curve; DKI, Diffusion kurtosis imaging; DWI, Diffusion weighted imaging; ICC, Interclass correlation coefficient; IVIM, Intravoxel incoherent motion; MRI, Magnetic resonance imaging; ROC, Receiver operating characteristic; ROI, Regions of interest.
